# ELD-YOLO: A Lightweight Framework for Detecting Occluded Mandarin Fruits in Plant Research

**DOI:** 10.3390/plants14111729

**Published:** 2025-06-05

**Authors:** Xianyao Wang, Yutong Huang, Siyu Wei, Weize Xu, Xiangsen Zhu, Jiong Mu, Xiaoyan Chen

**Affiliations:** 1 College of Information Engineering, Sichuan Agriculture University, Ya’an 625014, China; 202205858@stu.sicau.edu.cn (X.W.); 202205961@stu.sicau.edu.cn (Y.H.); 202207797@stu.sicau.edu.cn (W.X.); 202308556@stu.sicau.edu.cn (X.Z.); jmu@sicau.edu.cn (J.M.); 2 College of Computer and Information Engineering, Tianjin Normal University, Tianjin 300387, China; siyuwei008@gmail.com

**Keywords:** mandarin, complex orchard environment, fruit detection, edge feature, machine vision

## Abstract

Mandarin fruit detection provides crucial technical support for yield prediction and the precise identification and harvesting of mandarin fruits. However, challenges such as occlusion from leaves or branches, the presence of small or partially visible fruits, and limitations in model efficiency pose significant obstacles in a complex orchard environment. To tackle these issues, we propose ELD-YOLO, a lightweight detection framework designed to enhance edge detail preservation and improve the detection of small and occluded fruits. Our method incorporates edge-aware processing to strengthen feature representation, introduces a streamlined detection head that balances accuracy with computational cost, and employs an adaptive upsampling strategy to minimize information loss during feature scaling. Experiments on a mandarin fruit dataset show that ELD-YOLO achieves a precision of 89.7%, a recall of 83.7%, an mAP@50 of 92.1%, and an mAP@50:95 of 68.6% while reducing the parameter count by 15.4% compared with the baseline. These results demonstrate that ELD-YOLO provides an effective and efficient solution for fruit detection in complex orchard scenarios.

## 1. Introduction

Mandarins are rich in various vitamins and nutrients and particularly have high vitamin C content; each 100 g of mandarin pulp contains 53.2 mg of vitamin C. Their nutritional value and unique flavor make them one of the most consumed fruit categories worldwide [1]. China is the largest mandarin producer globally, accounting for 28.3% of the world’s total production and 30% of the total cultivated area. Despite its large-scale operations, the mandarin harvesting sector faces challenges such as seasonal labor shortages, increasing labor costs, and low harvesting efficiency [2]. To address these issues, the industry is accelerating the mechanization and intelligent transformation of harvesting operations [3]. Against this backdrop, precise fruit detection technology has become a core technical barrier in the development of intelligent harvesting equipment [4].

Traditional fruit detection relies on manually extracting fruit features such as texture and color, which are easily affected by subjective judgment and environmental interference [5]. The emergence and development of deep learning have ushered fruit detection into a new phase. By using an end-to-end image processing approach, deep learning can directly output detection results and continuously optimize model performance as the data scale increases. Due to its outstanding performance, deep learning has become the primary method for addressing fruit detection challenges [6].

In this context, many high-performance detection networks have been proposed and applied to fruit detection. These networks offer excellent scalability and can meet the demands of most fruit detection tasks. For example, ref. [7] proposed the YOLOv4 Drone model, which introduces lightweight feature enhancement and multi-scale fusion strategies based on the YOLOv4 architecture, making fruit detection via drones feasible. UAV-based YOLO detection frameworks have also been extended to other complex scenarios, such as wind turbine crack inspection under motion blur conditions, further validating the adaptability and robustness of YOLO in real-world visual tasks beyond static conditions [8]. However, due to limitations in handling small or occluded targets, this model inevitably suffers from a decline in detection accuracy. Ref. [9] proposed the YOLO-P model, which combines inverted shuffle blocks and Convolutional Block Attention Module (CBAM) attention mechanisms to improve the accuracy and speed of plum detection in complex backgrounds Although the inverted shuffle blocks optimize computational efficiency through channel reorganization, their feature reuse mechanism has limitations in adapting to multi-scale occlusions. Ref. [10] introduced the MSOAR-YOLOv10 model, which integrates multi-scale feature fusion and mixed loss strategies to improve apple detection in complex orchard environments. However, it was found, in practical deployment, that its multi-branch design increases memory access frequency, affecting the stability of real-time detection.

As can be seen from the above, most studies have yet to provide effective solutions to the challenges of severe occlusion and weak feature representation for small objects in complex orchard environments. The first challenge is occlusion. In complex orchard scenes, the dense distribution of fruit targets and tree backgrounds results in composite occlusions caused by leaf cover, fruit overlap, and background interference, which significantly hinder detection accuracy. The primary challenges in complex orchard environments are occlusion and small-object detection, both of which are closely associated with the issue of edge degradation. In densely distributed orchard scenes, occlusions caused by leaf cover, fruit overlap, and background interference often result in blurred or distorted object contours, making it difficult to distinguish object boundaries [11]. Existing methods, such as those employing CBAM or multi-scale fusion strategies, aim to enhance feature saliency but are typically insensitive to fine-grained edge details, especially in overlapping regions. This leads to a loss of structural cues that are critical to accurate localization. On the other hand, small objects inherently possess weak and fragmented edge features. When further occluded, these features are easily overwhelmed by noise or background clutter. However, current detection networks lack explicit mechanisms to perceive such degraded boundaries, resulting in reduced robustness and detection accuracy under real-world orchard conditions.

To solve these problems, this study proposes the ELD-YOLO model, an improved version of the YOLO11 model for mandarin detection in complex orchard environments. The main contributions of this study are as follows:

1. A dataset of 2388 images was established, covering mandarin orchards under various weather and lighting conditions, in different occlusion scenarios, and against diverse backgrounds.
2. A multi-scale edge feature enhancement module, Edge-guided Multi-scale Dual-domain Enhancement (EMD), was proposed and embedded into the C3K2 layer. An edge feature fusion structure (EAFF) was designed to construct complementary high-frequency features, enabling the comprehensive extraction of edge features.
3. A lightweight task-interactive detection head (LIDH) was designed, combining group convolution with an IoU-based task interaction mechanism. This reduces parameters while decreasing the false-positive and false-negative rates for occluded and small targets, achieving lightweight and efficient detection.
4. The dynamic point sampling module (Dysample) was introduced to replace the original upsampling structure. This enhances the model’s ability to handle densely distributed fruit, preserves features of occluded and small mandarins, and reduces the parameter count.

## 2. Related Work

### 2.1. You Only Look Once

The You Only Look Once (YOLO) series of algorithms represents a benchmark in single-stage object detection. In 2016, ref. [12] introduced the first YOLO model, which employed an end-to-end architecture capable of real-time detection at 45 Frames Per Second (FPS), with a lightweight structure that facilitated cross-domain transfer learning. In 2017, YOLOv2 improved recall and localization accuracy by introducing anchor-based detection and optimizing anchors via K-means clustering. YOLOv3 [13], released in 2018, replaced the backbone with Darknet-53, incorporated feature pyramid networks (FPNs) [14] for enhanced multi-scale detection, and switched to logistic regression for classification to balance speed and accuracy. In 2020, YOLOv5 restructured training on a PyTorch 2.3.0 framework, improving inference efficiency. YOLOv8 [15], introduced in 2023, further refined network architecture, introduced adaptive learning rate strategies, and redesigned detection heads to achieve a better accuracy–efficiency tradeoff. Most recently, in 2024, YOLO11 [16] replaced YOLOv8’s C2f module with the C3K2 module, using smaller 2 × 2 convolution kernels to enhance local feature extraction while reducing computation. It also added a C2PSA module in the neck network, combining multi-head attention with feedforward networks to better capture important features. These advances collectively improve detection accuracy, speed, and computational cost, motivating our selection of YOLO11 as the baseline.

### 2.2. Fruit Object Detection

Before the emergence of deep learning, fruit detection primarily relied on traditional image processing techniques. These methods identified key fruit characteristics by applying color space transformations and morphological filtering. They combined these with handcrafted features and classifiers and subsequently localized objects by using sliding windows or region proposal techniques. While intuitive, these methods struggled to adapt to complex scenarios, resulting in poor detection accuracy and robustness [17].

With the rise of deep learning, particularly the success of convolutional neural networks (CNNs) [18] in image classification, fruit detection shifted toward deep learning-based methods [19]. Models such as Faster R-CNN and Mask R-CNN [20] used region proposal networks (RPNs) to generate candidate regions before performing classification and regression, significantly improving detection accuracy. For example, ref. [21] applied Faster R-CNN to detect apples and mangoes in orchards, achieving substantial accuracy improvements. However, the high computational complexity of two-stage detectors makes them unsuitable for real-time detection in agricultural applications.

To balance detection accuracy and speed, research has increasingly focused on single-stage detectors, which leverage end-to-end architectures to significantly enhance inference speed. As a leading approach, the YOLO series has gained prominence in fruit detection due to its superior balance between speed and accuracy. In 2019, ref. [22] proposed MangoYOLO, which combined YOLOv3’s multi-scale detection capabilities with YOLOv2 (tiny)’s lightweight design, achieving an F1 score of 0.89 and real-time performance of 14 FPS in mango orchard scenarios. In 2020, ref. [23] developed LedNet, marking a breakthrough in feature enhancement techniques. This model integrated a feature pyramid network (FPN) to strengthen multi-scale feature fusion and utilized dilated spatial pyramid pooling to expand the receptive field, achieving 85.3% accuracy and 82.1% recall in dense apple occlusion scenarios.

To address complex orchard background interference, ref. [24] (2023) proposed YOLOv5s-FP for pear detection, achieving strong visual performance and robustness to occlusion and lighting variations. The model excelled in detecting pears of various sizes in dense, overlapping, and poorly lit environments, reaching a highest AP of 96.12%. In 2025, ref. [25] proposed a lightweight detection method, YOLO-CiHFC, to address challenges such as similar background color, leaf occlusion, high fruit density, and small fruit in orchard environments. By replacing all C2f modules in the YOLOv8n backbone and neck with Committed Information Rate (CiR) modules, the model maintained low complexity and parameter count while enhancing feature extraction and fusion. YOLO-CiHFC achieved an F1 score of 85.95%, an AP of 88.00%, and the smallest model size of 3.15 MB, delivering optimal detection performance across various scenarios.

These studies not only validate the strong adaptability of the YOLO framework in fruit detection but also provide valuable references and technical guidelines for future model improvements.

## 3. Results

### 3.1. Comparison Experiments

To validate the effectiveness of the algorithm improvements, this paper conducts a horizontal comparison between ELD-YOLO and versions of the YOLO series, classic detection models such as SSD [26] and Faster R-CNN, and the lightweight detection model NanoDet. As shown in Table 1, by comparing seven core metrics—F1 score, precision, recall, mAP@50, mAP@50:95, parameter count, and FPS—it is observed that although the detection accuracy of other YOLO versions is similar to the baseline model YOLO11, there are clear disadvantages in model complexity control. Specifically, while YOLOv7t [27] achieves a 0.4% improvement in mAP@50 over the baseline model YOLO11, its parameter count increases by 3.81 M. The SSD model excels in precision but reveals weaknesses in its anchor box mechanism due to a severe imbalance between the recall rate (65.1%) and mAP@50:95 (50.1%). Faster R-CNN, due to its two-stage detection architecture, falls behind single-stage detectors in both F1 score and mAP@50:95. NanoDet-Plus, as a lightweight single-stage detector, achieves a high inference speed of 205.09 FPS with only 4.30 million parameters. However, it suffers from a low recall rate of 63.6%, leading to an imbalanced F1 score of 79.0%. Its performance in small-object detection is further limited, as indicated by a relatively low mAP. Therefore, the ELD-YOLO composite model selected in this paper outperforms the other models across all metrics, achieving the highest detection capability with the fewest model parameters.

**Figure 1:**
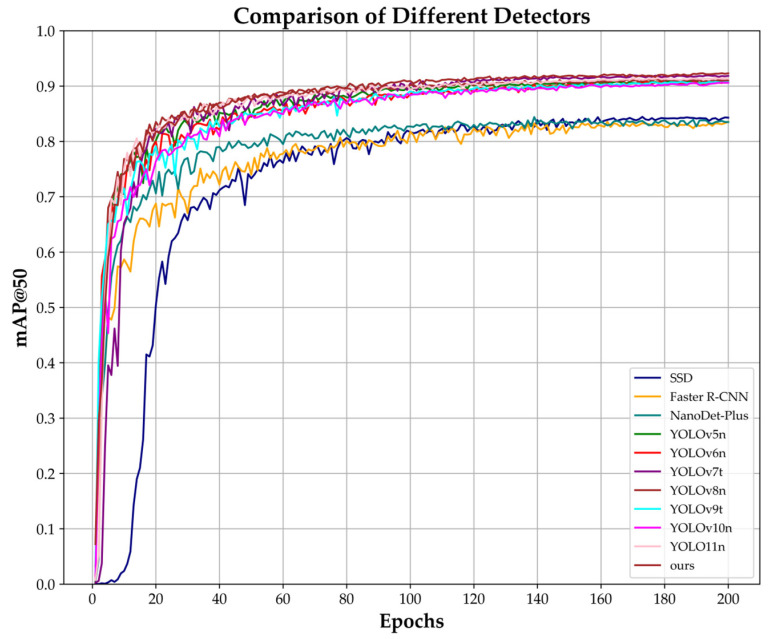
Variations in accuracy for mAP@50 during the training process of different detectors.

Figure 1 was plotted by comparing the changes in mAP@50 during the training process. As shown in the figure, ELD-YOLO outperforms the other models in terms of both convergence speed and accuracy, further validating its detection capability.

### 3.2. Ablation Study

The ELD-YOLO model proposed in this paper is based on YOLO11 and explores the impact of three improvement modules: the EMD module, the LIDH module, and the Dysample module, hereinafter referred to as E, L, and D, respectively. The model is evaluated by using F1 score, precision, recall, mAP@50, mAP@50:95, and parameter count as quantifiable metrics. To ensure robustness and account for performance variability, each ablation experiment was repeated five times with different random seeds. The results reported in Table 2 represent the averages and standard deviations, both rounded to two decimal places.

The analysis shows that after introducing module E into YOLO11, the model’s precision increased by 0.6%, but the recall did not show significant improvement. This indicates that enhancing the model’s edge feature perception contributes to improved precision. When module L was further added, the model’s recall improved by 0.8%, and the F1 score, mAP50, and mAP50:95 all showed notable increases, rising by 1.0%, 0.8%, and 0.8%, respectively. At the same time, the parameter count was further reduced to 2.19 M. This demonstrates that the lightweight task interaction detection head design not only reduces model complexity but also enhances the model’s ability to detect small objects. After integrating module D into the ELD-YOLO model, the parameter count increased by only 0.01 M compared with YOLO11+E+L. However, both mAP50 and mAP50:95 showed further improvements, reaching 92.1% and 68.7%, respectively. This confirms that the Dysample upsampling module’s excellent feature reconstruction capability contributes significantly to the model’s detection performance. Overall, ELD-YOLO achieves a significant improvement in detection accuracy while reducing the parameter count compared with the original model, making it highly effective for mandarin detection tasks in complex orchard environments.

### 3.3. Comparison with YOLO11

**Figure 2:**
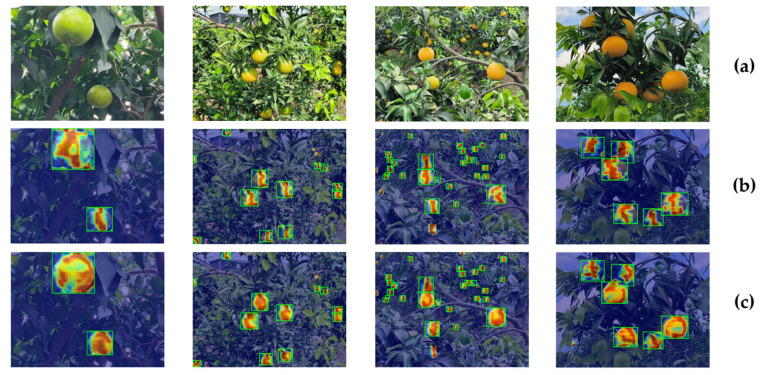
The visualization examples generated by GradCAM++. (**a**) Original images. (**b**) Results of YOLO11. (**c**) Results of ELD-YOLO.

To better highlight the improvements made by ELD-YOLO compared with YOLO11, we used Grad-CAM++ [28] to generate heatmaps for a visual analysis of the model, as shown in Figure 2. From the visualization results, it can be seen that the heatmap of ELD-YOLO is more complete and better aligns with the features. This clearly demonstrates that ELD-YOLO has superior detection capabilities compared with YOLO11. The following research results will focus on two major areas, i.e., occluded-object detection and small-object detection, to more intuitively showcase the improvements of ELD-YOLO over YOLO11.

#### 3.3.1. Comparison of Occluded-Object Detection Capability

**Figure 3:**
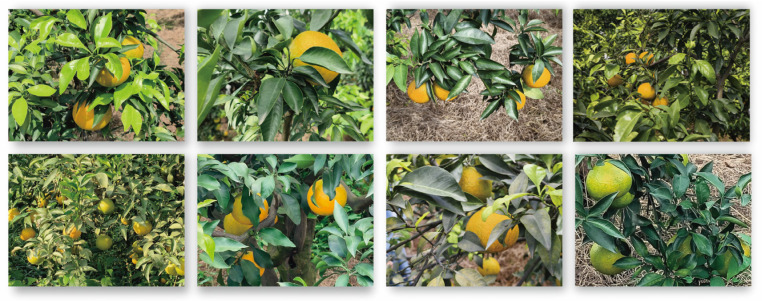
Illustrative examples from the High-Occlusion Subset.

Occluded objects pose a significant challenge in orchard detection. To quantitatively evaluate the model’s robustness to occlusion, we constructed a High-Occlusion Subset consisting of 83 images selected from the main dataset, where the majority of mandarin fruits are occluded by more than 50%. Figure 3 shows several examples from this subset.

The detection metrics on this subset demonstrate that ELD-YOLO significantly outperforms the baseline YOLO11 model under heavy occlusion conditions, as shown in Table 3. ELD-YOLO achieves a precision of 89.9 percent compared with 88.7 percent for YOLO11, a recall of 83.0 percent versus 82.8 percent, and an mAP at an IoU of 0.50 of 92.0 percent against 90.5 percent. Furthermore, the mAP across IoU thresholds from 0.50 to 0.95 improves to 68.1 percent, exceeding YOLO11’s 66.2 percent, indicating enhanced robustness and accuracy in detecting occluded fruits.

**Figure 4:**
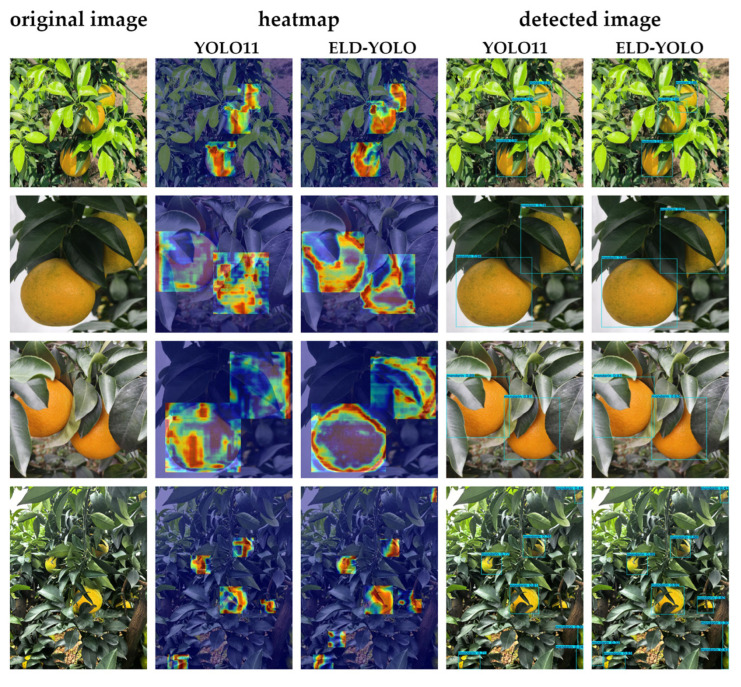
Comparison of occluded-object detection between ELD-YOLO and YOLO11.

In addition to these quantitative results, we also conducted qualitative comparisons of detection heatmaps and prediction confidence between ELD-YOLO and YOLO11. As shown in Figure 4, ELD-YOLO effectively identifies occlusions and focuses on the edge features of the fruits. In terms of prediction confidence, ELD-YOLO demonstrates a higher level of certainty for occluded fruits compared with YOLO11. This proves that ELD-YOLO has superior occluded-object detection capabilities and validates the feasibility of the edge feature enhancement approach.

#### 3.3.2. Comparison of Small-Object Detection Capability

**Figure 5:**
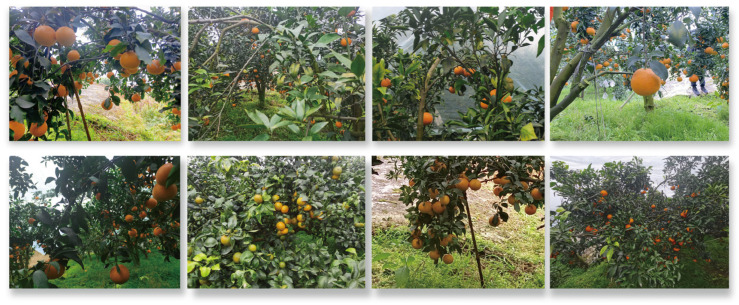
Illustrative examples from the Small-Object Mandarin Dataset.

Due to their small size, small-target mandarin fruits are often prone to missed detection. With ELD-YOLO, we enhanced its ability to detect small-target fruits by constructing a task-interactive detection head. To validate the optimization effect of ELD-YOLO on small-target detection, we built a Small-Object Mandarin Dataset, as shown in Figure 5.

This dataset is another set captured by the team in a mandarin orchard and was not used for model training [29]. It contains 40 long-range images to ensure a sufficient number of small-target mandarin fruits. A comparative experiment between ELD-YOLO and YOLO11 was conducted on this dataset. As shown in Table 4, ELD-YOLO achieves a higher recall of 91.2 percent compared with the 86.4 percent obtained by YOLO11, indicating a stronger ability to detect small and easily overlooked targets. Slight improvements in precision and mAP are also observed, reflecting overall enhanced performance in small-object instances.

**Figure 6:**
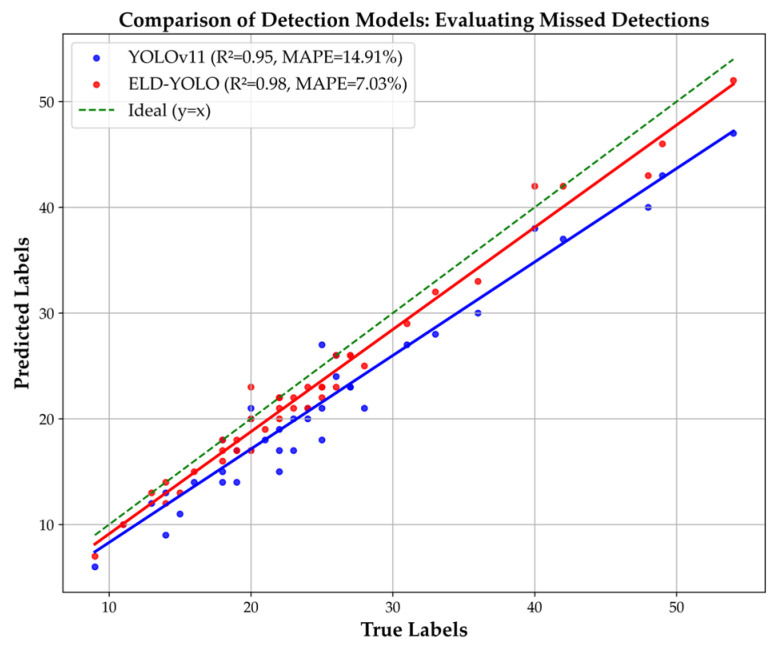
Linear regression plot of ELD-YOLO and YOLO11.

By performing a statistical analysis of the detection results of both models on this dataset, as shown in Figure 6, it is clearly evident that the linear regression equation (red line) of ELD-YOLO fits better with the ground truth (green line) than YOLO11 (blue line). The correlation index R of ELD-YOLO is 98%, which is 3% higher than YOLO11’s correlation coefficient R; the mean absolute percentage error (MAPE) is 7.03%, which is 7.88% lower than YOLO11’s MAPE [30]. This proves that ELD-YOLO has a lower miss detection rate than YOLO11 and exhibits superior small-target detection capabilities.

### 3.4. Applicability

To evaluate the generalization capability of the ELD-YOLO and YOLO11 models on other fruit types, we utilized the dataset provided by [31] (2019) from the MinneApple benchmark, which contains orchard images of apples captured under varying density and lighting conditions. The dataset was split into training, validation, and test sets in a 7:2:1 ratio. As shown in Table 5, both YOLO11 and ELD-YOLO achieved competitive performance on the apple detection task.

Table 5 demonstrates that the ELD-YOLO model proposed by the team achieves strong performance on this dataset. Compared with YOLO11, ELD-YOLO shows improvements in precision, recall, and mAP@50, reaching 85.0%, 76.7%, and 85.0%, respectively. The higher recall indicates that ELD-YOLO has better capability to handle missed detections, especially in complex orchard environments with occlusions and dense fruit clusters. Moreover, ELD-YOLO attains a faster inference speed of 128.83 FPS, outperforming YOLO11’s 112.27 FPS. These metrics further confirm its efficiency and robustness, underscoring the model’s potential for fruit detection tasks that demand both high accuracy and real-time performance.

## 4. Materials and Methods

### 4.1. Dataset Construction

#### 4.1.1. Data Acquisition

Building a suitable dataset is a prerequisite for evaluating model performance. In this study, we conducted a field investigation in late October in Dongpo District, Meishan City, Sichuan Province—one of China’s renowned orchard regions [32]. During the mandarin fruit-bearing season, we captured 2388 images of mandarin orchards by using a Canon R-10 and a Huawei Mate 60, with image resolutions of 6000 × 4000 and 3072 × 4096, respectively. These images contained a total of 19,494 mandarin fruits.

To replicate realistic and challenging orchard environments, images were captured under various weather conditions, including sunny and overcast days, covering three different time periods: morning, noon, and afternoon. To enhance visual diversity, various camera angles and distances were employed to capture mandarin fruits exhibiting differences in color, size, background complexity, maturity level, degree of overlap, and extent of leaf occlusion. The dataset includes three widely cultivated mandarin varieties in Meishan, i.e., Ehime mandarin (Aiwen Gan), Bapa mandarin (Baba Gan), and Wogan, each of which has unique morphological characteristics. All images were acquired during the fruit enlargement and ripening stages, which are critical to object detection due to increased fruit density, high occlusion rates, and strong visual similarity between fruits and surrounding foliage during these phenological phases.

**Figure 7:**
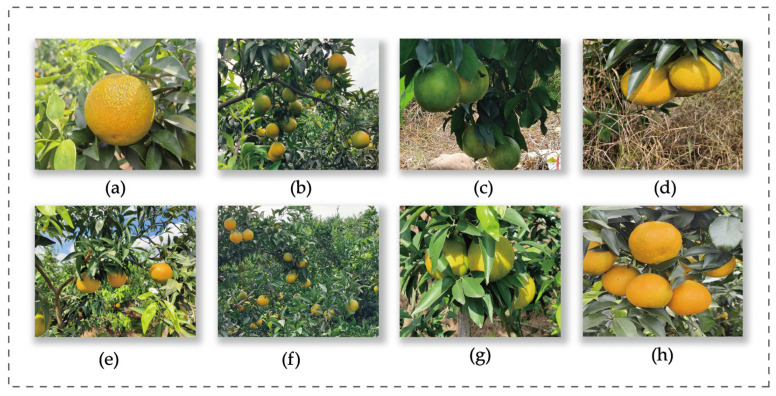
Part of the dataset is shown. (**a**) Single fruit. (**b**) Multiple fruits. (**c**) Unripe fruits. (**d**) Ripe fruits. (**e**) Natural light. (**f**) Low light. (**g**) Foliage-obscured fruits. (**h**) Overlapping fruits.

Figure 7 illustrates a set of mandarin images captured in a typical complex environment. The collected 2388 images were randomly and independently divided into training, validation, and test sets in a ratio of 7:2:1. Table 6 presents the dataset split details and the number of annotated objects.

#### 4.1.2. Dataset Preprocessing

In this study, we used the LabelImg [33] annotation tool to construct the object detection dataset, naming the fruit label “mandarin” and strictly following the YOLO format for data storage. The annotation task was assigned to six individuals: four annotators independently performed the labeling, while two additional annotators jointly reviewed the annotations to ensure quality and consistency. Discrepancies were discussed and resolved to maintain a high standard of annotation accuracy. The annotation process adhered to the following standards:

1. The bounding box must tightly fit the visible contour of the fruit.
2. Occluded fruits should be annotated by restoring their maximum possible contour.
3. Fruits with over 90% occlusion or bounding boxes shorter than 5 pixels must not be annotated.

Considering that the YOLO framework inherently integrates various data augmentation techniques—including basic spatial transformations, color adjustments, and composite strategies such as mosaic and mixup—we did not apply additional local augmentations to the dataset. This approach prevents model overfitting while preserving the authenticity of the dataset distribution. By leveraging the built-in augmentation mechanisms of YOLO, we avoided redundant enhancements that could introduce data bias.

### 4.2. ELD-YOLO

Orchard detection requires the comprehensive consideration of both model accuracy and computational efficiency. The YOLO series has long been known for its excellent detection speed and end-to-end training process. YOLO11 inherits the advantages of the YOLO series while further improving detection accuracy and robustness through network architecture optimization. Through experiments on the collected mandarin dataset, YOLO11 demonstrates strong adaptability compared with other models, particularly in complex background environments, where it can effectively handle both regression and classification tasks. Moreover, YOLO11 also offers computational efficiency advantages, allowing it to be effectively deployed on resource-constrained devices, thereby meeting real-time detection requirements. Considering the model’s accuracy and parameters, YOLO11 was ultimately chosen as the base model for this study.

**Figure 8:**
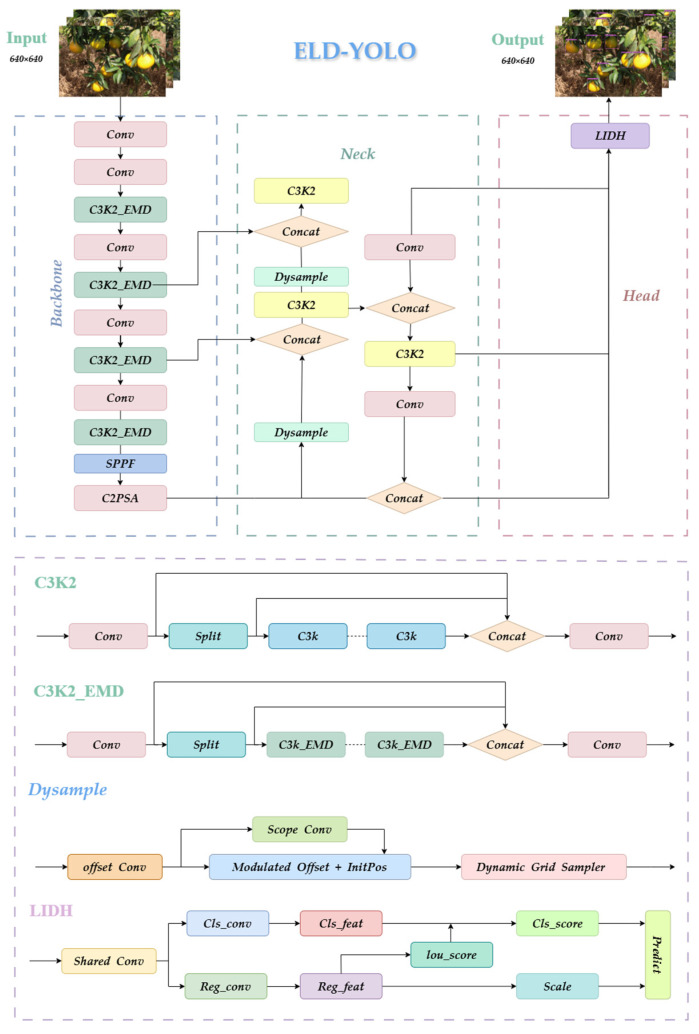
Model architecture of ELD-YOLO.

However, when dealing with mandarin detection tasks in complex orchard environments, YOLO11 still has certain limitations. For instance, mandarin fruits are often densely clustered on trees, overlapping with each other, leaves, and branches. In such cases, YOLO11 may make mistakes during detection, especially when objects are very close or completely overlapping. Additionally, due to the large number of mandarin fruits on fruit trees and the limited shooting distance, the dataset contains many small mandarin targets, which YOLO11 may struggle to detect effectively, resulting in missed detections. To address these issues, the study proposes ELD-YOLO, whose model structure is shown in Figure 8.

ELD-YOLO consists of three parts: the backbone, the neck, and the head. In the backbone, ELD-YOLO introduces the EMD module to enhance the edge features in the image and embeds them into the C3K2 module, which optimizes the model’s ability to detect occluded and small objects. In the head, ELD-YOLO replaces the original detection head with LIDH, which reduces the number of parameters through group convolution and introduces a task interaction module to enhance feature expression for occluded and small objects, achieving efficient and accurate orchard mandarin detection. Additionally, ELD-YOLO replaces the upsampling module in the neck network with Dysample to improve the model’s ability to detect dense objects.

#### 4.2.1. C3K2-EMD

**Figure 9:**
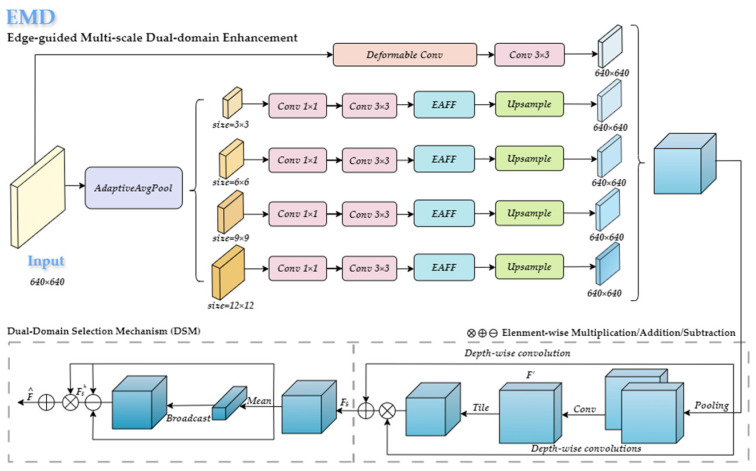
Structure of EMD module.

In fruit detection in occlusion scenarios, edge features play a decisive role in the accuracy of target localization; due to occlusion, the semantic integrity of the target is compromised, and the generation of detection boxes heavily relies on contour features. Edge semantics offer better guidance for task regression than internal semantics, which are prone to interference. Based on this understanding, this study proposes an Edge-guided Multi-scale Dual-domain Enhancement (EMD) module. This module consists of multi-scale edge feature enhancement and a dual-domain feature selection mechanism, as shown in Figure 9.

**Figure 10:**
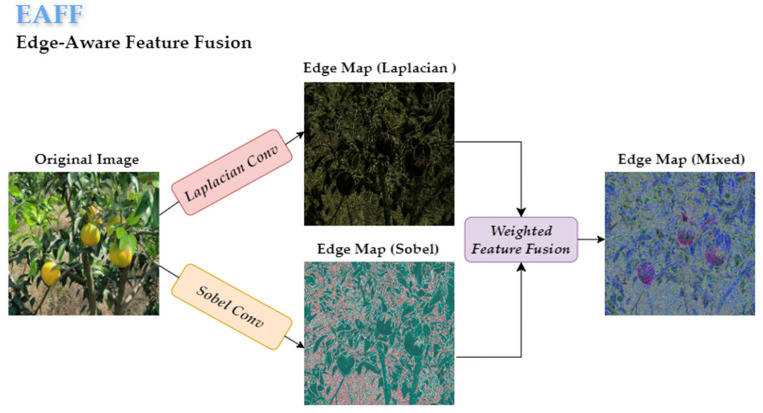
Structure of the EAFF.

The multi-scale edge feature enhancement module strengthens edge features through a dual-path heterogeneous processing approach. The auxiliary path uses deformable convolution [34] for geometrically adaptive feature extraction, retaining the target’s original spatial information. The main path generates a multi-scale feature pyramid through adaptive average pooling, which is then compressed by convolution and input into the edge enhancement unit (EAFF). Within the EAFF, the Sobel operator (first-order gradient filtering) and the Laplacian operator (second-order derivative filtering) are used in parallel to extract direction-independent fine details and direction-sensitive gradient edges. These are fused with a weighted combination to construct complementary high-frequency features, as shown in Figure 10. The features processed by the main path are concatenated again with the auxiliary path features via a residual connection. This residual fusion not only preserves the edge structure but also facilitates effective cross-scale information integration, enabling the network to capture both fine-grained local cues and high-level semantics. The EMD module first enhances the edge details at the feature level and then merges cross-scale information at the path level, ultimately generating a multi-scale feature output ***F*** that combines semantic integrity and edge sharpening.

For the final output feature ***F*** after feature concatenation, this study introduces a dual-domain selection mechanism [35]. This mechanism combines depthwise separable convolutions with a dynamic spatial gate (DSM SpatialGate) to enhance the edge contour features of the fruit, effectively suppressing background interference from branches and leaves while highlighting mandarin edge information. The enhanced spatial feature is computed as

(1) $$Y={DWConv}_{1}\left(X\right)\cdot \sigma \left({Conv}_{\mathrm{spataal}}\left(ChannelPool\left(X\right)\right)\right)+{DWConv}_{2}(Y),$$

where DWConv_1_ and DWConv_2_ are depthwise convolutions with different dilation rates and σ denotes the sigmoid activation applied to the spatial attention weights derived from pooled features. In parallel, a local feature calibration module based on mean difference, called DSM LocalAttention, is introduced. This module adaptively reweights channels to enhance the detailed response of small and occluded targets, such as texture features of partially hidden fruits in leaf gaps. The attention mechanism is formulated as

(2) $$F=a\cdot \left(X-\mu \right)\cdot X+b\cdot X,$$

where X is the input feature map, μ is the mean of X over spatial dimensions, and a, b ∈ R^C×1×1^ are learnable channel-wise parameters.

Finally, dynamic parameters are used to balance the intensity of spatial enhancement and local refinement, enabling the adaptive optimization of features based on occlusion levels and target scale differences. This effectively addresses core challenges in orchard scenarios such as blurred edges and the attenuation of small features by providing more discriminative representations for the detection head.

By cascading multiple C3K2-EMD layers, the proposed method constructs a cross-scale edge feature pyramid, reinforcing the representation ability of micro-edge features (shallow layers) and semantic edges (deep layers) at different depths of the backbone network. This enables the comprehensive multi-scale extraction of edge features.

To assess the individual contributions of the sub-components within the proposed C3K2-EMD module, we conducted a series of component-wise ablation experiments. As shown in Table 7, we systematically removed key components such as the EAFF unit and the dual-domain selection mechanism. In particular, we also performed ablation on the Sobel and Laplacian operations within the EAFF module to assess their individual contributions. The results indicate that each component plays a positive role in enhancing detection performance, thereby validating the effectiveness of the overall module design.

#### 4.2.2. LIDH

In complex orchard environments, occluded and small objects are often affected by their small size, which impacts their classification score (cls_score), leading to misdetection. Additionally, the complexity of orchard environments places certain demands on model lightweighting. To reduce missed detections and achieve a lightweight design for the detection head, the study innovatively proposes the lightweight task interaction detection head (LIDH). The LIDH reduces the number of parameters by constructing a lightweight feature-sharing structure through group convolutions and designs a task interaction module. This module enhances the classification score of small-sized targets by using the IoU scores obtained from the regression task, thus reducing missed detections.

**Figure 11:**
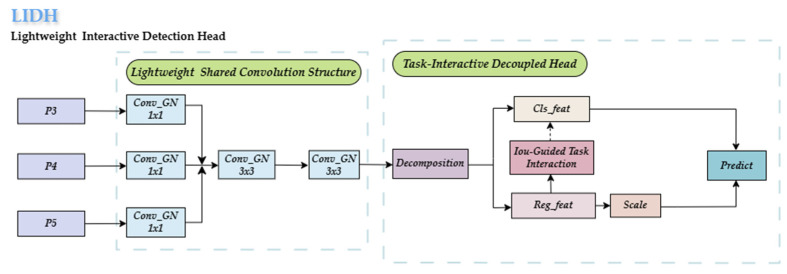
Structure of LIDH module.

The structure of the LIDH is shown in Figure 11. The LIDH consists of two core components: a lightweight shared convolution structure [36] and a feature–task interaction structure. The lightweight shared convolution module uses a staged processing flow: First, it applies 1 × 1 group convolutions to adjust the channel dimensions and align features across multiple scale feature layers (P3, P4, and P5). Then, the processed feature tensors are cross-scale-concatenated and fused. Finally, a 3 × 3 group convolution is used to extract deep features and share them across the fused features. This hierarchical feature processing mechanism using group convolutions effectively extracts and shares low-level features, providing the foundation for subsequent collaborative optimization between tasks.

The feature–task interaction structure of the LIDH first decouples the detection tasks to enable differentiated feature extraction and then enhances the detection of occluded and small targets through an IoU-based cross-task optimization mechanism. Since small targets occupy limited spatial regions, even slight localization deviations can cause a significant drop in the IoU, leading to training instability [37]. Inspired by this observation, we explore how the IoU can be leveraged to guide classification for small and occluded targets. Although these targets often yield low IoU values, their actual positional errors may be negligible.

To address this, we propose an IoU-aware cross-task optimization mechanism that uses localization cues from the regression branches to refine classification confidence, thereby reducing the likelihood of misclassifying true targets as background. The workflow is as follows: The classification branch first generates initial classification confidence scores (cls_feat) through convolution followed by sigmoid activation. In parallel, the regression branch utilizes deformable convolutions to enhance localization features and then applies a 1 × 1 convolution to produce the predicted IoU score. Finally, the classification confidence is dynamically adjusted by combining cls_feat and the predicted IoU through a weighting strategy, as defined by the following formula:

(3) $$w=\sigma \left(\mathrm{IoU}\;\mathrm{score}-0.5\right)\;\times \;1\left(\mathrm{IoU}\;\mathrm{score}\;<\;0.5\right)$$

(4) $$\mathrm{cl}s_{\_}\mathrm{feat}=\mathrm{cl}s_{\_}\mathrm{feat}\;\times \;\left(1+w\right)$$

where w is the enhancement weight and σ is the sigmoid function, while 1 is an indicator function that returns 1 if the IoU score is less than 0.5 and 0 otherwise. This design compensates for the classification confidence of small and occluded targets with low IoU scores, reducing the missed detection rate, while avoiding excessive enhancement of high-IoU-score targets, thus preventing false detections. It effectively prevents missed detections caused by the decay of classification confidence (cls_feat) for small-scale and occluded targets in complex orchard detection scenarios. Experimental results show that the LIDH not only reduces the number of model parameters but also effectively improves detection accuracy and recall, validating the effectiveness of the interaction mechanism between classification and regression tasks.

To validate the effectiveness of the proposed LIDH module, we conducted a comprehensive comparison with representative detection heads, including YOLO11, DyHead, and SEAMHead. As presented in Table 8, the LIDH’s precision is 88.9%, and the recall is 83.4%, slightly below DyHead’s 89.2% precision and 83,6% recall. However, the LIDH achieves the smallest model size at 2.18 million parameters and the fastest speed of 226.08 FPS. These results show that LIDH balances accuracy and efficiency well for real-time orchard detection.

#### 4.2.3. Dysample

**Figure 12:**
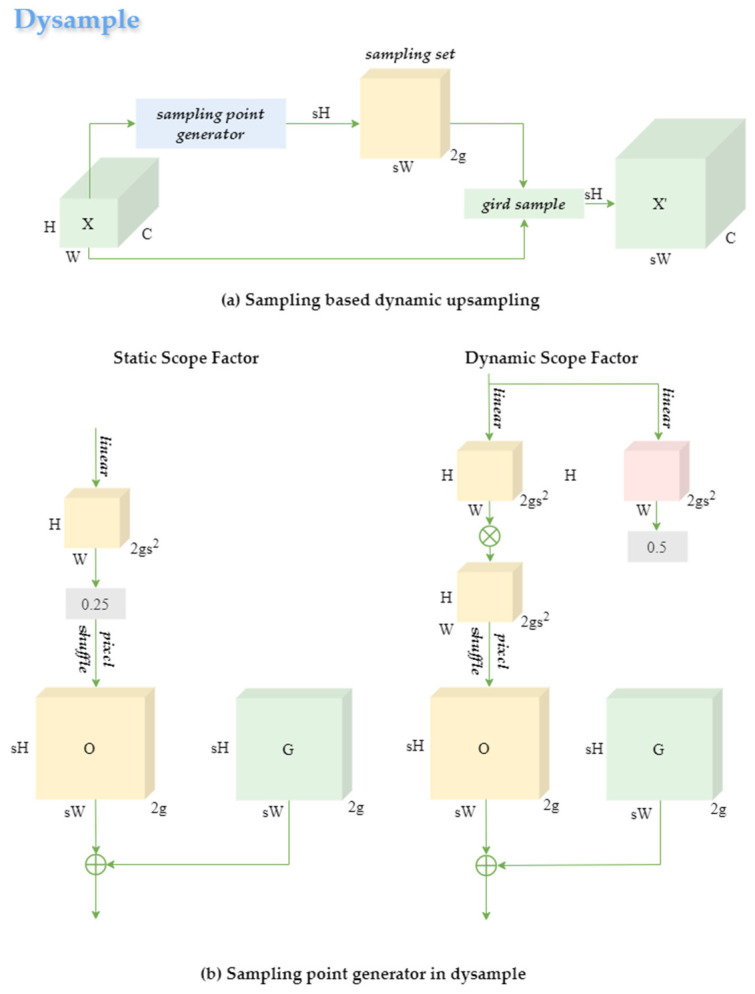
Structure of Dysample.

In complex orchard scenes, dense canopy growth and mutual occlusion among fruits lead to a significant reduction in visual features for small or partially hidden oranges. Traditional upsampling methods often degrade these features further due to interpolation artifacts. To mitigate this issue, our approach integrates Dysample [38], a dynamic point sampling module, into the upsampling stage of the neck network, replacing conventional upsampling operations, as shown in Figure 12.

Dysample performs upsampling by directly resampling feature points rather than relying on dynamic convolution or bilinear interpolation. This strategy enhances the resolution and fidelity of fine details while reducing computational cost. Specifically, given an input feature map x, a sampling set S is generated via a sampling point generator. The output $x^{\prime }$ is then obtained by applying PyTorch’s grid_sample function:

(5) S=G+O

(6) $$x^{\prime }={\mathrm{grid}}_{\_}\mathrm{sample}\left(x,S\right)$$

Here, G denotes the original regular sampling grid, and O is the offset generated by a combination of static and dynamic range factors. The static offset component $O_{1}$ is defined as

(7) $$O_{1}=0.25\left[\mathrm{linear}\left(x\right)\right]$$

The dynamic offset component $O_{2}$, modulated through sigmoid-like control, is formulated as

(8) $$O_{2}=0.5\sigma \left[\mathrm{linear}1\left(x\right)\right]\cdot \mathrm{linear}1\left(x\right)$$

The final sampling offset O aggregates these contributions. This design enables adaptive sampling grid adjustment guided by both low-level structure and high-level semantics, effectively capturing edge details and enhancing small-object regions.

As illustrated in Figure 8, Dysample enhances the resolution of feature maps in the neck, making small and occluded fruits more distinguishable. The module’s “Pixel Shuffle + Linear” (PL) offset strategy notably reduces parameter count compared with the traditional “Linear + Pixel Shuffle” (LP) method, without sacrificing accuracy.

To assess the impact of different upsampling strategies on detection performance and efficiency, we replaced the Dysample module with three widely used alternatives: the original upsampling module used in the baseline, Transposed Convolution, and CARAFE. Table 9 summarizes the comparison on the mandarin fruit dataset. It can be observed that integrating the Dysample module leads to notable improvements in recall, precision, and FPS. Although its mAP@50 is slightly lower than that of CARAFE, Dysample demonstrates clear advantages in terms of model size and real-time detection performance. These results highlight the effectiveness of the dynamic point sampling strategy and confirm the superiority of Dysample as an upsampling method in complex orchard scenarios.

### 4.3. Evaluation Metrics

To comprehensively evaluate the detection model’s capability to recognize mandarin fruits in real orchard operational scenarios, this study establishes a multi-dimensional analysis approach, adopting precision (P), recall (R), F1 score, and mean average precision (mAP) as core quantitative metrics. While these metrics are widely used in object detection, their behavior can vary significantly under certain conditions, such as class imbalance, occlusion, and small-object detection, and it is important to consider these factors when interpreting the results.

Precision (P) is defined as the ratio of correctly predicted positive samples to all predicted positive samples. This metric effectively reflects the model’s likelihood of falsely identifying background interference as mandarin. Higher precision indicates a lower false-positive rate and better overall performance. However, it does not account for missed detections, which are particularly problematic in crowded, occluded environments where fruits overlap. The specific calculation is shown in Equation (9), where TP denotes the number of correctly identified mandarin instances and FP represents the number of background elements incorrectly classified as mandarin.

(9) $$\mathrm{Precision}=\frac{\mathrm{TP}}{\mathrm{TP}+\mathrm{FP}}$$

Recall (R) measures the proportion of actual mandarin targets that are correctly detected, quantifying the model’s risk of missed detections in complex orchard and forestry environments. In cases of severe occlusion or small-object detection, recall may be underestimated because the model may detect large and visible fruits while failing to identify occluded or small ones. This highlights the need to balance recall with other metrics to avoid false assurances of completeness. The formula is shown in Equation (10), where TP is the number of correctly identified mandarin instances and FN refers to mandarin targets missed due to factors such as occlusion by foliage or long-distance shooting.

(10) $$\mathrm{Recall}=\frac{\mathrm{TP}}{\mathrm{TP}+\mathrm{FN}}$$

The F1 score, shown in Equation (11), represents the harmonic mean of precision and recall, offering a comprehensive evaluation of a model’s performance by balancing both metrics.

(11) $$F1\;\mathrm{score}=2\;\times \;\frac{\mathrm{Precision}\;\times \;\mathrm{Recall}}{\mathrm{Precision}+\mathrm{Recall}}$$

Average precision (AP) is a key metric for evaluating the performance of a model on a single class. It captures the overall detection ability by balancing precision and recall across different confidence thresholds. mAP generalizes this to multi-class scenarios, calculated as the arithmetic mean of AP values for all mandarin varieties, thus reflecting the model’s generalization capability. In mandarin detection tasks, the mean AP at an IoU threshold of 0.5 (mAP@50) serves as a basic performance benchmark, while the mean AP under progressively increasing IoU thresholds from 0.5 to 0.95 (mAP@50:95) provides a systematic evaluation of the model’s sensitivity to mandarin shape variations and localization accuracy. The relevant calculation formulas are defined as follows:

(12) $$\mathrm{AP}=\int_{0}^{1}P\left(r\right)\mathrm{dr}$$

(13) $$\mathrm{mAP}=\frac{1}{N}\sum_{i=1}^{N}AP_{i}$$

In Equation (12), the variable represents precision, referring to the precision value corresponding to different recall thresholds on the precision–recall curve. The integral operation, calculated as the area under the curve, globally quantifies the detection performance. In Equation (13), N denotes the total number of mandarin subspecies categories, and $AP_{i}$ represents the average precision for the i-th mandarin category. This evaluation metric system effectively reveals the model’s practical detection capability under typical orchard challenges such as uneven lighting and occlusion interference.

### 4.4. Training Environment and Experimental Setup

To ensure the fairness and reproducibility of model training and evaluation, all experiments in this study were conducted under a unified experimental environment and fixed hyperparameter configurations, thereby avoiding interference in results caused by environmental differences or parameter fluctuations. The experimental environment and hyperparameter settings in this study are shown in Table 10.

Before finalizing the hyperparameter values, we conducted preliminary experiments to identify configurations that led to stable convergence and good performance. The final choices, including 200 training epochs, a batch size of 32, a learning rate of 0.01, and momentum of 0.9, were found to yield the best results in terms of convergence speed and model generalization. The loss curves consistently converged within the training schedule, confirming the adequacy of these settings for effective model training.

## 5. Discussion

The ELD-YOLO model, through the integration of an edge-aware module and a task-interactive detection head, demonstrates excellent performance in key metrics such as detection accuracy. It effectively alleviates challenges related to occlusion and small-object detection in mandarin orchard scenarios. The innovative design of this model provides a valuable technical reference for other agricultural fruit detection tasks. However, this study still has several limitations. Future work will focus on the following aspects:

1. Although the current edge-aware detection scheme effectively handles typical occlusions, its performance still needs improvement in camouflaged environments where the target and background share highly similar features. We hypothesize that the introduction of contrastive learning mechanisms may help disentangle target features from background noise, but this assumption still requires empirical validation through dedicated, stratified datasets, which will be a key focus of future research.
2. The IoU-based feature enhancement method for small objects has successfully reduced missed detections, but it may increase false positives against complex backgrounds. Future research will explore more discriminative feature interaction strategies, integrating multi-dimensional information to better balance missed and false detections.
3. Due to constraints in data collection, current experiments are limited to a few mandarin varieties and lack the validation of generalization to other types, such as Tangelo and Kumquat. Future work will focus on creating a dataset that includes multiple varieties of mandarin and various growth stages. Additionally, we will leverage transfer learning to enhance the model’s adaptability to diverse visual characteristics across different species.

## 6. Conclusions

This paper proposes ELD-YOLO, an edge-aware lightweight object detection framework designed to address the complex challenges of citrus fruit detection in orchard environments. By incorporating a multi-scale edge enhancement module (EMD), a lightweight task-interactive detection head (LIDH), and a dynamic point upsampling strategy (Dysample), the model significantly improves detection accuracy in scenarios involving occlusion and small objects while maintaining a compact architecture with only 2.2M parameters. Experimental results based on a custom-built orchard dataset show that ELD-YOLO outperforms existing mainstream models in terms of precision (89.7%), recall (83.7%), and mAP metrics.

Despite its strong quantitative performance, the model still has certain limitations. The current evaluation is restricted to specific mandarin varieties and environmental conditions, and its generalization ability across other citrus types, such as Tangelo and Kumquat, as well as under highly heterogeneous conditions like varying illumination, weather, and soil backgrounds, remains untested. Future work will focus on building a more diverse dataset covering multiple citrus species and phenological stages and validating the model under a broader range of environmental conditions. In terms of practical deployment, we also plan to explore implementing ELD-YOLO on embedded platforms such as agricultural robots or UAVs to evaluate its performance and energy efficiency tradeoffs in real-world operations.

In conclusion, ELD-YOLO provides an effective solution for high-precision and lightweight citrus detection in complex orchard environments. This work lays a solid foundation for developing scalable and adaptive fruit detection models in the context of smart agriculture.

## Figures and Tables

**Table 1:** Results of the comparison study.

Model	F1 (%)	Precision (%)	Recall (%)	mAP@50 (%)	mAP@50:95 (%)	Parameters (M)	FPS
YOLO11	86	88.6	82.6	90.7	66.3	2.58	217.74
YOLOv10n	85	87.3	81.3	90.2	66.1	2.71	230.35
YOLOv9t	85	88.4	81.5	90.1	66.4	2.01	137.38
YOLOv8n	86	88.5	82.6	90.5	66.8	3.01	188.42
YOLOv7t	86	87.2	83.7	91.1	64.8	6.01	157.10
YOLOv6n	86	88.4	82.1	90.3	65.9	4.24	191.63
YOLOv5n	86	88.1	82.2	90.2	66.3	2.50	160.43
NanoDet-plus	79	81.1	63.6	80.3	49.6	4.30	205.09
SSD	75	89.8	65.1	82.8	50.1	23.80	65.32
Faster R-CNN	72	71.3	73.8	80.2	44.0	4.38	39.42
**ELD-YOLO**	**88**	**89.8**	**83.7**	**92.1**	**68.7**	**2.20**	**235.34**

**Table 2:** Results of the ablation study.

Method	F1 (%)	Precision (%)	Recall (%)	mAP@50 (%)	mAP@50:95 (%)	Parameters (M)	FPS
YOLO11	86	88.62 ± 0.13	82.58 ± 0.21	90.67 ± 0.09	66.34 ± 0.12	2.58	217.74
YOLO11+E	86	89.21 ± 0.09	82.56 ± 0.11	91.41 ± 0.17	66.69 ± 0.23	2.59	209.21
YOLO11+E+L	87	89.67 ± 0.12	83.41 ± 0.09	91.64 ± 0.12	67.53 ± 0.07	2.19	218.32
YOLO11+E+L+D	88	89.77 ± 0.14	83.71 ± 0.15	92.12 ± 0.17	68.69 ± 0.14	2.20	235.34

**Table 3:** Results on the High-Occlusion Subset for ELD-YOLO and YOLO11.

Method	Precision (%)	Recall (%)	mAP@50 (%)	mAP@50–95 (%)
YOLO11	88.7	82.8	90.5	66.2
ELD-YOLO	89.9	83.0	92.0	68.1

**Table 4:** Results on the Small-Object Mandarin Dataset for ELD-YOLO and YOLO11.

Method	Precision (%)	Recall (%)	mAP@50 (%)	mAP@50–95 (%)
YOLO11	88.2	86.4	88.7	56.3
ELD-YOLO	90.1	91.2	91.2	59.9

**Table 5:** Results of ELD-YOLO and YOLO11 on the MinneApple Dataset.

Method	Precision (%)	Recall (%)	mAP@50 (%)	mAP@50:95 (%)	Parameters (M)	FPS
YOLO11	84.1	75.6	84.9	43.2	2.58	112.27
ELD-YOLO	85.0	76.7	85.0	43.8	2.20	128.83

**Table 6:** The division of the dataset.

Name	Proportion (%)	Number of Pictures	Number of Labeled Mandarin Fruits
Training	70	1671	13,120
Validation	20	478	4291
Test	10	239	2083
Total	100	2388	19,494

**Table 7:** Results of the ablation study on components of the C3K2-EMD module.

Methods		Precision (%)	Recall (%)	mAP@50 (%)	mAP@50:95 (%)	Parameters (M)	FPS
YOLO11		88.6	82.6	90.7	66.3	2.58	217.74
Full C3K2-EMD		89.2	82.4	91.3	66.7	2.59	209.21
*w*/*o* EAFF		88.9	82.3	90.9	66.5	2.58	211.47
*w*/*o* DSM	*w*/*o* Sobel	88.5	82.5	90.9	66.4	2.53	216.24
	*w*/*o* Laplacian	88.8	82.4	91.0	66.3	2.53	216.51
	Full EAFF	89.0	82.6	91.2	66.6	2.53	216.90

**Table 8:** Results of LIDH compared with other detection heads.

Method	Precision (%)	Recall (%)	mAP@50 (%)	mAP@50:95 (%)	Parameters (M)	FPS
YOLO11	88.6	82.6	90.7	66.3	2.58	217.74
DyHead	89.2	83.6	91.0	67.4	3.08	197.32
SEAMHead	88.7	82.9	90.6	66.6	2.50	220.19
LIDH	88.9	83.4	91.1	67.3	2.18	226.08

**Table 9:** Results of Dysample compared with other upsampling methods.

Methods	Precision (%)	Recall (%)	mAP@50 (%)	mAP@50:95 (%)	Parameters (M)	FPS
YOLO11	88.6	82.6	90.7	66.3	2.58	217.74
CARAFE	89.1	82.6	91.2	67.1	2.72	160.74
Transposed Convolution	88.4	82.8	90.8	66.8	2.61	212.11
Dysample	89.0	83.2	91.1	67.3	2.59	240.12

**Table 10:** Experimental environment and model parameters.

Environment	Version	Parameter	Value
Python version	Python 3.9	Initial Learning Rate	0.01
PyTorch version	PyTorch 2.3.0	Num_workers	8
Operating system	Ubuntu 20.04	Batch size	32
CUDA version	CUDA 12.1	Epochs	200
Central Processing Unit	Intel Xeon Platinum 8352V	Momentum	0.9
Graphics Processing Unit	NVIDIA GeForce RTX 3080x2	Image size	640 × 640

## Data Availability

The dataset used in this study is part of ongoing research and is not publicly available due to its continued use in related experiments. Researchers with specific needs may contact the corresponding author for further information.
